# Italian immunization calendar: rationale and schedule

**DOI:** 10.1186/1824-7288-41-S1-A12

**Published:** 2015-09-24

**Authors:** Gabutti Giovanni, Kuhdari Parvanè, Stefanati Armando

**Affiliations:** 1 Department of Medical Sciences, University of Ferrara, Ferrara, Italy

## 

Vaccines are a great scientific conquest of the modern era having made it possible to prevent many infectious diseases that previously had a significant impact on the population both in terms of morbidity and sequelae and/or lethality. They act respecting/enhancing physiological capabilities of the organism and have a very favorable pharmacoeconomic profile. The development and availability of a safe, well-tolerated and effective vaccine is the indispensable premise for the primary prevention through vaccination in the community. Then it is necessary to define the target to be pursued; this latter can be control, elimination and eradication. The choice of the target is influenced by several factors; however, it must be followed by the development of an appropriate strategy to achieve the necessary vaccine coverage rate. The vaccination schedule is therefore the instrument by which each vaccine strategy is planned, and should take into account some epidemiological, immunological and practical requirements (Table 1) [1]. The immunization schedule is therefore the chronological sequence of each vaccination; it is an essential tool to achieve established targets as well as a useful guide for any stakeholder (e.g. health care professionals, users, ect.). The current vaccine schedule results from a series of laws relating to compulsory vaccinations and has been integrated over time by including recommended immunizations [2]. A key point is that the schedule should be a flexible instrument and continuously updated. The Italian immunization schedule has many positive aspects as it is updated based on the availability of new vaccines, combines mandatory and recommended immunizations (stressing in this way the relevance of both types of vaccinations), is structured so that it can be integrated in case of new epidemiological requirements and covers all age groups. Besides, it includes vaccines considered of priority from the public health point of view and for this free of charge and actively offered.

**Table 1:** Immunizations schedule: main factors that should be taken into account

Epidemiological factors • Typical age of acquistion of the disease (immunization should come first) • Possible age-related complications of the disease • Age-related adverse events of immunizaton • Already implemented immunization programs
**Immunological factors** • Immune system development • Clearance of maternal antibodies • Number of doses and time interval between doses necessary to obtain an effective immune response • Duration of immune protection elicited by immunization

**Practical factors** • Number of immunizations to be included in the schedule • Availability of combined vaccines • Number of vaccination sessions • Organization of immunization services

Concerning the paediatric age, particularly the first year of life, the definition of the schedule should take into account the development of the immune system, the clearance of maternal antibodies, the typical age of acquisition of infection, the possible age-related complications of a disease, the possible side effects of immunization according to age of immunization, the duration of elicited immune protection, the number of appointments for vaccination sessions and the accessibility to vaccination services. The scientific societies SItI and SIP, togheter with FIMP and FIMMG, has approved the “2014 Lifetime Immunization Schedule” (Table 2) [3].

**Table 2:** 2014 Lifetime Immunization Schedule up to 15 months of age (SItI, SIP, FIMP, FIMMG) (modified from ref. 3)

Vaccine	0-30 days	3 months	4 months	5 months	6 months	7 months	11 months	13 months	15 months
**DTPa**		DTaP		DTaP			DTaP^		

**IPV**		IPV		IPV			IPV^		

**HBV**	HBV*	HBV		HBV			HBV		

**Hib**		Hib		Hib			Hib		

**PCV**		PCV13		PCV13			PCV13	PCV13^^	

**MMRV**								MMRV§	

**MMR**								MMR§	

**Varicella**									V§

**Men C**								conjugate Men C** or Men ACWY**	

**Men B**		Men B	Men B		Men B			Men B§§	

**HPV**									

**Influenza**						Flu°°			

**Rotavirus**		Rotavirus##							

**HAV**									HAV##

^ a fourth dose has to be provided at 5-6 years of age; for the fourth dose dTap could be used instead of DTaP if a high coverage rate is guaranteed

* newborns from HBaAg positive mother; co-administration of IgG at birth

^^ children starting immunization during the second year of life should receive two doses. One dose in the case of immunization started in the third year of life. One dose of PCV13 is recommended for unimmunized children or for those who have been immunized with PCV7. Two doses are recommended for children at risk

§ a second dose has to be provided at 5-6 years of age

** a second dose is recommended at 12-14 years of age: in subjects at risk immunization could start at 3 months of age (3rd dose after one year of age)

§§ schedule 3+1 in newborns; the fourth dose at 13-15 months of age

°° subjects at risk and/or attending kindergarten or communities

## recommended as universal vaccination. Could be co-administered to any other immunization of the first months of life
